# Social support in early-stage breast cancer patients with fatigue

**DOI:** 10.1186/s12905-020-01106-2

**Published:** 2020-10-29

**Authors:** Hege Lilleskare Sørensen, Tore Kr. Schjølberg, Milada Cvancarova Småstuen, Inger Utne

**Affiliations:** 1 Department of Otorhinolaryngology, Head and Neck Surgery, Division for Head, Neck and Reconstructive Surgery, Oslo University Hospital, Oslo, Norway; 2 Department of Nursing and Health Promotion, Faculty of Health Sciences, OsloMet – Oslo Metropolitan University, St. Olavs plass, Postbox 4, 0131 Oslo, Norway

**Keywords:** Breast cancer, Social support, Cancer-related fatigue, Cross-sectional study

## Abstract

**Background:**

A large number of women with breast cancer suffer from fatigue, and social support is described as having a positive impact on health in stressful life situations. The aim of this study is to evaluate social support in a sample of early-stage breast cancer outpatients with fatigue during treatment, and to evaluate the association between cancer-related fatigue and social support and between social support and demographic and treatment characteristics.

**Method:**

This cross-sectional study includes 160 outpatients with early-stage breast cancer and cancer-related fatigue. The patients were recruited from clinics at a university-based cancer centre in Norway. The research instruments included The Social Provisions Scale (SPS), which measures ‛attachment’, ‛social integration’, ‛reassurance of worth’, and ‛nurturance’, and a fatigue questionnaire (FQ), which measures total, physical and mental fatigue. Data were analysed using descriptive statistics and linear regression analysis.

**Results:**

Median total score for SPS was 59 (min/max = 39/64). Significant associations were found between mental fatigue and the provisions ‛reassurance of worth’ (B = − 0.34, 95% CI = [− 0.60; − 0.08]) and ‛nurturance’ (B = 0.20, 95% CI = [0.08; 0.31]). In addition, an association was found between social support and living with someone (B = 6.09, 95% CI = [4.07; 8.11]). No associations were found between physical fatigue and social support or between social support and treatment variables.

**Conclusions:**

To a large extent, breast cancer patients with fatigue in this study experienced social support from their surroundings. The fact that there were significant associations between mental fatigue and two of the provisions of SPS suggests that social support is more closely related to mental fatigue than to physical fatigue. Findings from this study suggest that living with someone is important for the experience of social support during treatment for breast cancer. Clinicians need to evaluate demographic characteristics in relation to social support in early-stage breast cancer patients with fatigue.

## Background

Breast cancer is the most frequent form of cancer among women in Norway. In 2018 approximately 3,600 women were diagnosed, and the five-year relative survival rate is 90 per cent [1]. The prognosis of breast cancer depends on different factors, such as stage and grade of the tumor, and the relatively high survival rate is due to advances in cancer screening which enable early diagnosis [2]. However, the cancer treatment is demanding and protracted, entailing surgery, chemotherapy, radiation and targeted treatment in various combinations [2]. The improvements in screening, diagnostics and treatment have yielded increased recovery rates, but at the same time the complexities of treatment have resulted in challenges related to side effects [3].

Cancer-related fatigue (CRF) is the most common and most troublesome symptom among breast cancer patients undergoing treatment [4]. Various studies report that between 25 and 99 per cent of cancer patients experience CRF, and that it is at its worst during chemotherapy treatment [4–7]. CRF differs in nature from other manifestations of fatigue. In general, sleep or rest do not alleviate CRF, and the duration and severity is greater. In addition, CRF is often associated with high levels of distress and co-occurs with other symptoms such as pain, sleep disturbance and depression. CRF affects quality of life and reduces engagement in social activity and work [8].

Several factors are associated with how oncology patients experience CRF. Different demographics, medical status, and psychosocial and biological factors are associated with CRF [4]. For example, being single with low income is associated with higher level of fatigue in breast cancer patients receiving treatment, and fatigue during treatment has been shown to be negatively associated with a return to work [9, 10]. In addition, an Irish study of patients with moderate and serious fatigue during chemotherapy reports that receiving support from family and friends is the most commonly used self-management strategy against CRF [11].

Support from one′s surroundings has a positive effect on physical function, psychological well-being and the ability to adjust to living with cancer [12]. A Finnish longitudinal study shows that breast cancer patients who experience good social support decrease the risk of negative changes in quality of life at the start of their treatment trajectory. The most beneficial emotional support is mainly provided by the patient’s spouse, partner, children, siblings or friends [13]. This finding is supported by a Norwegian qualitative study of breast cancer patients [14]. These women report that the reassurance of friends, colleagues, health personnel, and most importantly from close family members, is their most important form of support. Physical presence, as well as knowing that someone is thinking of you, is significant.

Social support is understood and measured in a variety of ways in the literature [13, 15]. The theoretician Weiss has a multidimensional view of social support and presents several provisions which characterise a variety of relations that influence the experience of social support [16]. Based on this theory, Cutrona & Russell have developed an instrument, The Social Provisions Scale (SPS), which measures the degree of social support for the provisions ʻattachment’, ʻsocial integration’, ʻreassurance of worth’, ʻnurturance’, ʻguidance’ and ʻreliable alliance’. The provision ʻattachment’ is defined as a relationship that provides emotional closeness and a sense of security. ʻSocial integration’ is described as a sense of belonging to a group that shares similar interests. Recognitions of one's competence, skills and value describes the provision ʻreassurance of worth’. The provision ʻnurturance’ represents one's sense of responsibility for the well-being of others. ʻGuidance’ is provided in relationships with trustworthy individuals who can offer information and good advice. ʻReliable alliance’ is described as the assurance that others can be counted upon for tangible assistance [17].

The significance of, the mechanisms behind, and the understanding of social support in breast cancer patients undergoing treatment are not fully understood. However, a theoretical understanding indicates that good social support has a positive impact on health in stressful life situations [17]. Based on the notion that social support is essential for self-management among patients with breast cancer and CRF, the purpose of this study is to evaluate social support in early-stage breast cancer patients with CRF in the treatment phase. In addition, we want to report associations between CRF and social support, and between social support and demographic and treatment characteristics.

## Methods

### Sample and methods of data collection

This study is part of a randomised controlled trial evaluating a psycho-educational intervention to reduce patients’ CRF [18]. Recruitment took place in outpatient clinics at a university-based cancer centre in south-east Norway. This study is a cross-sectional descriptive study and includes baseline data from all patients before randomisation. At baseline the patients had received surgery, completed chemotherapy, were about to receive their final radiation therapy treatments (daily treatment number 25) and/or were in the first of five years of hormone therapy. To be included in the study, the women had to be undergoing active curative treatment for breast cancer stage I or II. In addition, as this was an intervention study for CRF, the women had to report a fatigue score of ≥ 2.5 on the numeric rating scale (NRS) (0–10 point variation). Eligible patients were ≥ 18 years of age, were able to read, write and understand Norwegian, and gave written informed consent. Eligible patients were approached in the outpatient clinics by the staff nurses. The women completed the self-reporting questionnaires at home and posted them back to the investigator [18]. There was no need to apply for license for any of the survey instruments used in this study.

### Instruments

#### Demographic and treatment characteristics

For demographic data, the patients reported their year of birth (year born), marital status (single, married/live-in partner, divorced, widow/widower or separated); living arrangements (living alone, living with spouse/partner, living with siblings, living with family/relatives, living with children/children-in-law, living with parents, living in an institution or living with others); educational level (primary school, secondary school, high school, university college or university); work situation (paid employment, self-employed, full-time home maker, student/military service, unemployed/laid off, on disability benefit or retired); type of breast surgery (removed lump in breast or whole breast) and treatment (chemotherapy, radiation, hormonal treatment or other).

#### Fatigue

Fatigue was measured using the fatigue questionnaire (FQ) which is translated into Norwegian [19]. The questionnaire consists of 11 questions, seven of which measure physical fatigue (PF) and four of which measure mental fatigue (MF). The questions have four response alternatives on a scale from 0 (less than usual) to 3 (much more than usual). The questions give a total score from 0 to 33 points, where a high score indicates more fatigue (total fatigue = TF). For physical fatigue (PF), the total score is reported on a scale from 1 to 21, and for MF from 0 to 12. FQ has good reliability and validity, and the questionnaire is used in Norwegian and in international studies [18–20]. This study has Cronbach’s alpha value for TF = 0.9, PF = 0.8 and MF = 0.8.

#### Social support

Social support is measured using the Norwegian version of SPS [21]. Six provisions were initially measured but, after revision, ʻguidance’ and ʻreliable alliance’ were removed due to intercorrelation [17, 22, 23]. The questionnaire therefore consists of 16 items with four items for each provision: ʻattachment’, ʻsocial integration’, ʻreassurance of worth’ and ʻnurturance’. The items have four response alternatives on a scale from 1 (strongly agree) to 4 (strongly disagree). Negative items yield the opposite point score. The total score can range from 16 to 64, where a high score indicates a greater level of social support. For the four provisions, the total score is reported on a scale from 4 to 16 [21]. The questionnaire is used in both Norwegian and international studies, and reports good reliability and validity [17, 21, 24]. This study has a Cronbach’s alpha value for total SPS score = 0.8; ʻattachment’ = 0.7, ʻsocial integration’ = 0.7, ʻreassurance of worth’ = 0.7 and ʻnurturance’ = 0.7.

#### Comorbidity

The self-administered comorbidity questionnaire (SCQ), translated into Norwegian, consists of 13 common medical conditions simplified into a language that can be understood without prior medical knowledge [25]. Patients indicated whether they had the condition; whether they received treatment for it (proxy for disease severity), and whether it limited their activities (indication of functional limitations). The patient can receive a maximum of three points for each condition. The total SCQ score ranges from 0 to 39. The SCQ has well established validity and reliability [26, 27].

### Statistical analyses

Descriptive statistics were used to describe demographic and treatment characteristics. Categorical variables are presented as counts with percentages. The continuous variables are presented with mean and standard deviation (SD) or with median and minimum and maximum values. The various demographic and treatment-related variables were dichotomised before further analyses. Univariate regression analysis was performed to find possible association between fatigue and social support, and a possible association between social support and demographic and treatment-related variables. As the variables living arrangement and marital status were strongly correlated with each other (*p* ≤ 0.05), only living arrangement was included in the regression analysis to avoid multicollinearity. The variables that were statistically significant in univariate analyses were further included in the multivariate regression analysis. *p* values < 0.05 were considered statistically significant. The statistics programme SPSS Statistics 23 for Windows was used to perform the analyses [28].

## Results

### Description of demographic and treatment characteristics and fatigue

A total of 415 women were asked to participate in the study, of which 149 were excluded because they had a fatigue score of < 2.5 on NRS. One hundred and six out of 266 patients declined to participate in the study. The final number of patients included in the study was 160, representing a response rate of 60.2% [18]. The baseline demographic, clinical characteristics, and fatigue scores of the patients are listed in Table 1. The mean age is 55.3 (SD = 9.4) years of age, ranging from 25 and 77 years. Half of the women have university college or university education. Most of the patients are married or cohabiting (70.3%), and 80.6 per cent live with somebody. The mean score for TF is 19.8 (SD = 4.6); PF 13.7 (SD = 3.2) and MF 6.1 (SD = 2.0). Approximately two-thirds (60%) have one or more comorbidities.

**Table 1:** Demographic and treatment characteristics, and fatigue scores for breast cancer patients with fatigue (n = 160)

Characteristics	n	%	Missing
*n*	160		
Age	160		0
49 years or younger	45	28.1	
50 years or older	115	71.9	
Number of comorbidities	96	60.0	3
One or two	62	38.0	
Three or more	34	22.0	
Marital status	158		2
Married/partner	111	70.3	
Single	47	29.7	
Living arrangement	155		5
Live alone	30	19.4	
Live with someone	125	80.6	
Education level	158		2
Primary, secondary, high school	79	50	
College university, university	79	50	
Work situation	158		2
Working	105	66.5	
Not working	53	33.5	
Surgical treatment	159		1
Remove whole breast	53	35.1	9
Remove only lump in breast	98	64.9	9
Chemotherapy	147		13
Yes	83	56.5	
No	64	43.5	
Radiation	160		0
Yes	158	98.8	
No	2	1.2	
Hormonal treatment	146		14
Yes	89	61	
No	57	39	

Fatigue		Average	SD
Total fatigue (0–33)	144	19.8	4.6
Physical fatigue (0–21)	145	13.7	3.2
Mental fatigue (0–12)	158	6.1	2.0

SD = standard deviation

### Social support scores

The median total score for social support is 59 and the minimum/maximum scores are 39 and 64 respectively. Median scores for the various provisions are: ʻattachment’ 16 (min/max = 8/16), ʻsocial integration’ 15 (min/max = 7/16), ʻreassurance of worth’ 16 (min/max = 9/16), and ʻnurturance’ 14 (min/max = 5/16).

### Association between social support and fatigue

Univariate analysis revealed a positive and statistically significant association between TF and ʻreassurance of worth’ (B = − 0.54, 95% CI = [− 1.03; − 0.41]). When ʻreassurance of worth’ increases by one point, TF will decrease by half a point, indicating a lower level of fatigue. There is also a positive association between MF and ʻattachment’, ʻreassurance of worth’ and ʻnurturance’. Multivariate analysis of MF and the significant provisions ʻattachment’, ʻreassurance of worth’ and ʻnurturance’ show significant association only between MF and ʻreassurance of worth’ (B = − 0.34, 95% CI = [− 0.60; − 0.08]) and between MF and ʻnurturance’ (B = 0.20, 95% CI = [0.08; 0.31]). Our data did not reveal any statistically significant association between PF and social support. See Table 2 for details.

**Table 2:** Linear regression analysis with social support and fatigue (n = 160)

	Univariate analysis			Multivariate analysis		
	B	95% CI	*p* value	B	95% CI	*p* value
*Total fatigue*						
SPS total	− 0.08	− 0.22; 0.07	0.302			
Attachment	− 0.43	− 0.93; 0.07	0.093			
Reassurance of worth	− 0.54	− 1.03; − 0.41	0.034*			
Nurturance	0.16	− 0.12; 0.44	0.252			
Social integration	− 0.21	− 0.66; 0.24	0.356			
*Physical fatigue*						
SPS total	− 0.04	− 0.14; 0.06	0.463			
Attachment	− 0.16	− 0.52; 0.19	0.368			
Reassurance of worth	− 0.16	− 0.51; 0.19	0.365			
Nurturance	0.05	0.14; 0.25	0.604			
Social integration	− 0.17	− 0.48; 0.15	0.301			
*Mental fatigue*						
SPS total	− 0.02	− 0.08; 0.44	0.580			
Attachment	− 0.23	− 0.44; − 0.02	0.034*	− 0.07	− 0.34; 0.20	0.594
Reassurance of worth	− 0.32	− 0.53; − 0.12	0.002*	− 0.34	− 0.60;− 0.08	0.011*
Nurturance	0.15	0.03; 0.27	0.011*	0.20	0.08; 0.31	0.001*
Social integration	− 0.04	− 0.23; 0.16	0.716			

*Level of significance < 0.05; CI = confidence interval; B = regression coefficient; SPS = The Social Provisions Scale; fatigue as the dependent variable; social support as the independent variable

### Association between demographic and treatment characteristics and social support

Univariate analysis revealed a strong and statistically significant association between living with someone and social support (B = 6.09, 95% CI = [4.07; 8.11]). No statistically significant association was found between social support and the other selected demographic variables or treatment variables (see Table 3).

**Table 3:** Linear regression analysis with demographic and treatment characteristics and social support (n = 160)

	Univariate analysis		
	B	95% CI	*p* value
*Demographic variables*			
Age Under/over 50 years	− 1.80	− 3.72; 0.13	0.067
Living arrangement With someone/alone	6.09	4.07; 8.11	0.000*
Educational level Primary, secondary, high school/collage university, university	1.32	− 0.43; 3.08	0.137
Employment Working/not working	1.39	− 0.46; 3.25	0.140
*Treatment variables*			
Surgery and radiation			
Other treatment Chemotherapy/hormonal therapy	0.79	− 1.34; 2.92	0.467

*Level of significance < 0.05; CI = confidence interval; B = regression coefficient; social support as the dependent variable; demographic characteristics, and treatment characteristics as the independent variables

## Discussion

This study is the first to evaluate social support using SPS during active treatment in early-stage breast cancer patients with CRF. The results show that the patients as a group experience social support from their surroundings and that social support is of significance for the experience of total and mental fatigue. Not surprisingly, living with someone has a positive impact on the women’s experience of social support.

The finding that the patients in this study of early-stage breast cancer patients report a high degree of social support is consistent with scores reported in a previous study of patients (n = 117) with suspected breast cancer awaiting diagnosis in Norway [29]. The SPS scores were quite similar, and the women in both studies report the lowest value for the provision ʻnurturance’. However, even if the women in these studies experience a high degree of social support, it is important to note that the variation in the degree of social support among the women was relatively large, in particular for the provision ʻnurturance’. These findings suggest that clinicians need to evaluate social support in the early phase of the breast cancer trajectory.

An important contribution of this study is that women with heavy responsibility for the nurturance of others are also the ones who experience a higher degree of mental fatigue. The finding that the women who care for others, which means that others are dependent on their help, care and consideration [17, 21], may indicate that these women need time for themselves to manage mental fatigue. This hypothesis is supported by a qualitative study of breast cancer patients waiting for surgical treatment which reported the importance of the possibility to be able to think only about oneself [14]. These women expressed that they needed a shoulder to cry on and not to be the carer. Women with heavy care responsibilities may be particularly vulnerable during treatment for early-stage breast cancer.

The women who received social support in relationships characterised by respect and value for their abilities and skills and by an appreciation of their knowledge (i.e., ‘reassurance of worth’) experienced a lower degree of mental fatigue. Of note, while 70% of the women in our sample are married or cohabiting and two-thirds are working, one should be cautious about drawing the conclusion that reassurance of worth is always provided by the partner. Findings from a study of early-stage breast cancer patients show that friends, children, siblings, colleagues and health personnel may also provide emotional support [13]. In addition, results from a cohort study (n = 2013) of recently diagnosed breast cancer patients, show that working increases self-worth, quality of life, a sense of meaning, and social integration [30]. These findings suggest that social roles that confirm one′s self-worth in the family, among friends or at work, is significant for the women’s experience of social support.

The lack of association between social support and physical fatigue suggests that social support is more closely related to mental fatigue than to physical fatigue. As noted in a review [31], most of the studies included found different correlates for physical and mental fatigue. As mental fatigue is characterised by the patient′s inability to find the right words to express what they want to do [4, 19, 32], it is important that the women receive social support which may help them to follow simple directions and retain the educational information that they need to care for themselves during treatment.

The results of our study show that living with someone is significant to the experience of social support for women with early-stage breast cancer and CRF. This finding is consistent with an Iranian study of women undergoing treatment for breast cancer [33]. These patients reported a positive correlation between being married and affective aspects of CRF [33]. The significance of living with someone is also emphasised in a qualitative study of women waiting for surgical treatment for breast cancer [14]. Having someone physically present and available at all times is described as important for the experience of social support [14].

It is important to point out that our sample consists of women with early-stage breast cancer with CRF. Thirty-six per cent of the relevant participants in the study were not included because they did not have fatigue at the time of inclusion [18]. It is therefore not surprising that the women in the sample report a high degree of fatigue. In this context it is interesting to note that a Norwegian study among non-selected women (not screened for fatigue) with breast cancer in the treatment phase reports a correspondingly high prevalence of fatigue [6]. Despite comparable results, we cannot generalise our findings to early-stage breast cancer patients in general. Approximately two-thirds of the women in our study had a comorbidity, and this may have influenced the level of CRF. As noted in an integrative review, there is an association between multiple chronic conditions and CRF; having one or more additional comorbidity was significantly associated with prevalence and severity of CRF [34].

### Strengths and limitations

The strength of this study is that the sample is relatively large and the age of the participants are representative of women with breast cancer in Norway.

The limitations of this study is the cross-sectional design, which does not provide causality, though association.

## Conclusions

This study is the first to assess social support in early-stage breast cancer patients with CRF using SPS while they are undergoing treatment. The fact that significant associations were found between mental fatigue and two of the provisions of SPS suggests that social support is more closely related to mental fatigue than to physical fatigue. Findings from this study suggest that living with someone is significant for the experience of social support during treatment for breast cancer. The findings of this study should be confirmed in a larger cross-sectional study as well as in longitudinal studies. In addition, it would be interesting to investigate how social support influence fatigue over time, as well as how fatigue influence social support over time.

## Data Availability

The datasets used and/or analysed during the current study are available from the corresponding author on reasonable request.

## Abbreviations

CRF: Cancer-related fatigue
SPS: Social provisions scale
FQ: Fatigue questionnaire
SCQ: Self-administered comorbidity questionnaire
TF: Total fatigue
PF: Physical fatigue
MF: Mental fatigue
NRS: Numeric rating scale
